# Cellular and Biophysical Pipeline for the Screening of Peroxisome Proliferator-Activated Receptor Beta/Delta Agonists: Avoiding False Positives

**DOI:** 10.1155/2018/3681590

**Published:** 2018-04-12

**Authors:** Natália Bernardi Videira, Fernanda Aparecida Heleno Batista, Artur Torres Cordeiro, Ana Carolina Migliorini Figueira

**Affiliations:** ^1^Brazilian Biosciences National Laboratory (LNBio), Brazilian Center for Research in Energy and Materials (CNPEM), 13083-970 Campinas, SP, Brazil; ^2^Graduate Program in Biosciences and Technology of Bioactive Products, Institute of Biology, State University of Campinas (Unicamp), Campinas, SP, Brazil

## Abstract

Peroxisome proliferator-activated receptor beta/delta (PPARß/*δ*) is considered a therapeutic target for metabolic disorders, cancer, and cardiovascular diseases. Here, we developed one pipeline for the screening of PPARß/*δ* agonists, which reduces the cost, time, and false-positive hits. The first step is an optimized 3-day long cellular transactivation assay based on reporter-gene technology, which is supported by automated liquid-handlers. This primary screening is followed by a confirmatory transactivation assay and by two biophysical validation methods (thermal shift assay (TSA) and (ANS) fluorescence quenching), which allow the calculation of the affinity constant, giving more information about the selected hits. All of the assays were validated using well-known commercial agonists providing trustworthy data. Furthermore, to validate and test this pipeline, we screened a natural extract library (560 extracts), and we found one plant extract that might be interesting for PPARß/*δ* modulation. In conclusion, our results suggested that we developed a cheaper and more robust pipeline that goes beyond the single activation screening, as it also evaluates PPARß/*δ* tertiary structure stabilization and the ligand affinity constant, selecting only molecules that directly bind to the receptor. Moreover, this approach might improve the effectiveness of the screening for agonists that target PPARß/*δ* for drug development.

## 1. Introduction

Peroxisome proliferator-activated receptor beta/delta (PPARß/*δ*) is a lipid-activated transcription factor, which is a member of the nuclear receptors (NR) superfamily that regulates the activation or silencing of several target genes. PPARß/*δ* is ubiquitously expressed in humans, although it is mainly found in the skin, placenta, brain, liver, kidneys, spleen, fat skeletal muscle, and digestive tube [1–3].

PPARß/*δ* is involved in some metabolic pathways such as energy metabolism, homeostasis, adipogenesis, and lipid metabolism [4–6]. Several studies have suggested that PPARß/*δ* modulation by agonists regulates food intake, body weight, insulin sensitivity, adiposity, and body mass [5, 7]. It has also been associated with diverse physiopathological processes, such as inflammation, obesity, dyslipidemia, diabetes, cancer, and cardiovascular diseases [6, 8–10]. PPARß/*δ* also has described extra-metabolic roles including neuroprotective effects against brain diseases, such as multiple sclerosis, strokes, Alzheimer's disease, and Parkinson's disease, and acts in cell differentiation and proliferation, immune regulation, oxidative stress, and skin biology [2, 3, 11].

The diversity in PPARß/*δ* function has been related to its ability to accommodate and bind different ligands in its ligand binding domain (LBD), with a wide range of natural and synthetic ligands. Among the natural ligands, there are fatty acids, prostaglandins, and leukotrienes [12, 13]. Several high affinity and subtype-specific PPARß/*δ* agonists have been developed and submitted for clinical trials for the treatment of metabolic diseases [1, 14]; however no ligand has been made available for clinical use.

Due to the high number of people affected by PPARß/*δ*-related disorders, the development of specific ligands to modulate the receptor activity becomes of great importance. Here, we developed and set up a suitable, cheaper, and robust screening pipeline for the better identification of PPARß/*δ* agonists. In the first step of this pipeline, we optimized the cell-based transactivation assay to be 1 to 2 days shorter and with the use of less reagents than the previously described ones, significantly reducing the costs in time and money for big screening campaigns. Additionally, we introduced two validation methods to avoid false positives: a thermal shift assay (TSA) to check PPARß/*δ* tertiary structure stabilization by the hit candidates, indicating direct binding to the protein, followed by an ANS fluorescence quenching assay to determine the compound/extract affinity for the PPARß/*δ* hydrophobic pocket.

To date, most of the screening methods for PPARs were based only on transactivation assays, which is the most common and well-established protocol to measure the activity of nuclear receptors [15–19]. However, this method may allow the selection of false-positive compounds that may activate PPARß/*δ* in an indirect way without agonist properties. To overcome this gap, we propose a pipeline in which the transactivation assay is followed by biophysical assays to confirm that the compound directly bound to the PPARß/*δ* ligand pocket.

Particularly, the major differences in our pipeline in comparison to other proposed PPARß/*δ* transactivation methods are the reduction of the assay length and volume; the cell carrier; automation; and the addition of biophysical validation methods [15–18]. Moreover, this pipeline was assessed by specific PPARß/*δ* agonists (GW0742, GW501516, and L-165,041) and the *Z*′-factor. In summary, we propose that this pipeline is a stable, cheaper, faster, and more robust tool to identify PPARß/*δ* agonists, and moreover, we tested a natural product library against the developed pipeline.

## 2. Material and Methods

### 2.1. Reagents

The materials for cell culture: Dulbecco's modified Eagle's medium (DMEM) was purchased from GIBCO Corporation (Carlsbad, CA, USA), fetal bovine serum (FBS) was obtained from CULTILAB (Campinas, SP, Brazil), and charcoal-stripped FBS and Penicillin/Streptomycin was obtained from GIBCO Corporation (Grand Island, NE, USA). Lipofectamine 2000® transfection reagent was obtained from Life Technologies (Indianapolis, IN, USA). The Dual Luciferase® Assay Reporter System was obtained from Promega Corporation (Madison, WI, USA). GW0742 (CAS 317318-84-6), GW501516 (CAS 317318-70-0), Bezafibrate (CAS41859-67-0), and L-165,041 (CAS 79558-09-1) were purchased from Sigma Corporation (St Louis, MO, USA).

### 2.2. Plasmids

The cell culture assays were performed after transfection of (i) pBIND-PPARß/*δ*, expressing Gal4-DBD and PPARß/*δ*-LBD fusion protein, and (ii) pGRE-LUC, which contains a Gal4 response element followed by firefly luciferase reporter gene. The PPARß/*δ*:GAL4 protein, when activated by a ligand, can induce the transcription of the luciferase reporter gene from pGRE-LUC. The pBIND-PPARß/*δ* modified vector and pGRE-LUC were a gift from Dr. Paul Webb (the Methodist Hospital Research Institute, Houston, EUA) [15, 17]. For transfection efficiency control (vector normalization), we used pRL-TK vector, which constitutively expresses* Renilla reniformis *luciferase [20, 21]. For* in vitro* protein assays, we used pET28a-His-LBD-PPAR*δ*, which contains the LBD gene of human PPARß/*δ* (aa 171-441) fused with His-tag [5].

### 2.3. Cell Culture

293T (ATCC® CRL-3216™) cells were maintained in DMEM supplemented with 10% (v/v) fetal bovine serum (FBS) and antibiotics (100 units/mL penicillin and 100 mg/mL streptomycin). Cells were grown in a 5% CO_2_, 95% air-humidified atmosphere, at 37°C.

### 2.4. PPARß/*δ* Transactivation Assay

**Figure 1:**
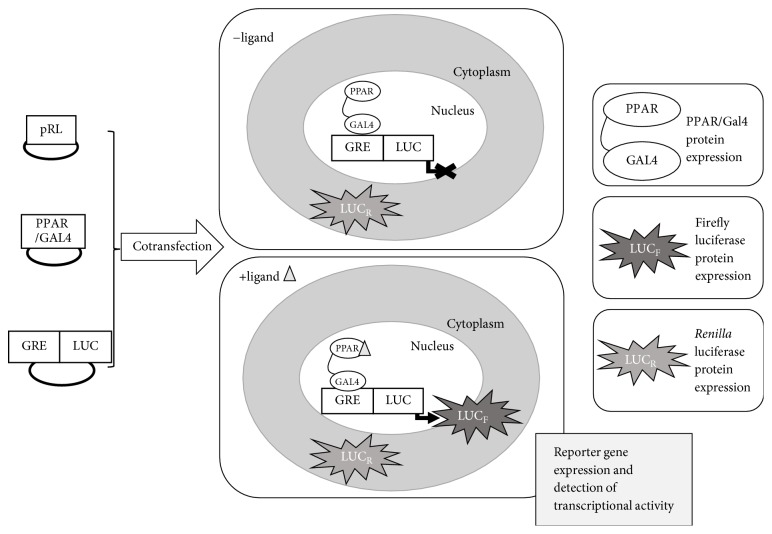
Principle of the* in vitro* PPARß/*δ* transactivation assay. This assay is based on the transient transfection of the 293T cell line with three plasmids: pBIND-PPARß/*δ* encoding a chimera of Gal4-DBD and PPARß/*δ*-LBD genes (PPAR/GAL4); pGRE-LUC, which owns one GAL4 response element upstream of a firefly luciferase reporter gene (LUC_F_); and the pRL vector, which constitutively express* Renilla *luciferase (LUC_R_). The transfected cells express both PPARß/*δ* and* Renilla *luciferase constitutively. When the transfected cell is exposed to a molecule that works as a ligand (+ligand), such as GW0742, PPARß/*δ* moves into the nucleus, binds to GRE-LUC, and triggers the expression of LUC_F_, which is the expected PPARß/*δ* activation effect in this assay. The reporter-gene expression correlates with the bioactivity of PPARß/*δ* in the sample.* Note.* For simplicity, only PPARß/*δ* monomer binding to GRE has been depicted.

The rationale of the primary screening assay is described in Figure 1. First, 293T cells at 70–90% confluence were transiently cotransfected with the plasmids pBIND-PPARß/*δ*, pGRE-LUC, and pRL in 100 × 20 mm plates with Lipofectamine 2000® transfection reagent, following the manufacturer's protocol. The transfected cells were incubated for 6 hours in a 5% CO_2_, 95% air-humidified atmosphere at 37°C.

In the meantime, the screening compounds (plant extracts) were reformatted to a 96-well plate (screening plates, white microplates, Perkin Elmer) containing 50 *μ*L of DMEM supplemented with 10% charcoal-stripped FBS at final concentration of 10 *μ*g/mL with Thermo Scientific™ Versette™ Automated Liquid Handler equipped with a 96-tip head. In each assay plate, column 1 was set up as negative control (vehicle, 1% dimethyl sulfoxide (DMSO)) and column 12 was set up as positive control (1 *μ*M GW0742, ~0.00047 *μ*g/mL).

After transfection, cells were seeded in 96-well white microplates (4 × 10^4^ cells per well), which already contained the controls or test compounds/extracts, in a final volume of 100 *μ*L per well of DMEM supplemented with 10% charcoal-stripped FBS and antibiotics (100 units/mL penicillin and 100 mg/mL streptomycin). Experimental conditions were adjusted to ensure linearity during the entire assay.

After 24 hours, the medium was aspirated with Thermo Scientific Versette Automated Liquid Handler, and luciferase activity was measured in each well with the Dual Luciferase Assay Reporter System (Promega). The reading solutions were added with Thermo Scientific Multidrop Combi Reagent Dispenser as follows: first, 20 *μ*L of lysis solution, followed by 20 min of plate incubation; then 25 *μ*L of LAR II substrate, followed by luminescence measurements in CLARIOstar® (BMG Labtech) plate reader; finally, 25 *μ*L of Stop&Glo® substrate was added and luminescence was measured.

We performed vector normalization with the raw luminescence data to control for differences in transfection efficiency between samples. For vector normalization we calculate luciferase signal/*Renilla *signal. The assay performance was assessed by plate statistics (signal-to-background ratio, *Z*′-factor, coefficient of variation) [22] and apparent cytotoxicity was a measure of* Renilla* luciferase parameters. For *Z*′-factor calculation, we consider, after vector normalization, the positive controls (GW0742) as signal, and negative controls (DMSO) as background. For cytotoxicity, wells with expression of* Renilla *reporter five standard deviations (5 × SD) below the mean value of the controls treatment were considered cytotoxic and consequently were disregarded. We considered as hit candidates compounds/extracts with luminescence* firefly/Renilla* ratio 7 times above the standard deviation of negative controls (DMSO) from the same plate. This last criterion takes in account the intrinsic variation of the negative controls signals by plate. In the screening graphics, the ratio* firefly/Renilla* for all the wells was normalized, positive control mean indicates 100% activation, and negative control mean was considered 0% activation. To confirm the hit candidates, all the selected extracts were retested in triplicate.

### 2.5. MTT Cytotoxicity Assay

Additionally, we related the 3-(4,5-dimethyl-2-thia-zoyl)-2,5-diphenyl-2H-tetrazolium bromide (MTT) results with* Renilla* reporter expression to assess when a test compound/extract might be toxic to the cells, only based on* Renilla *reporter expression. Then, 4 × 10^4^ 293T cells per well were seeded in a 96-well transparent microplates (Sarstedt) in 0.1%, 1%, 3%, 5%, 10%, and 50% DMSO and 1 *μ*M GW0742 and the general viability of the cells was determined by reduction of MTT to formazan [23]. After 20 hours of DMSO incubation, cell media were changed to phosphate buffer saline (PBS), and 10% MTT (5 mg/mL in PBS) was added to each well. Cells were incubated at 37°C, for 3 h, PBS was removed, and 100 *μ*L DMSO was added to dissolve the formazan crystals. The absorbance was measured at 562 nm using an EnSpire® Multimode Plate Reader (Perkin Elmer). The experiment was performed in 4 independent experiments. Data were analyzed by 2-way ANOVA followed by Sidak's multiple comparison test in GraphPad Prism software.

### 2.6. Protein Expression

The human PPARß/*δ* ligand binding domain (hPPARß/*δ* LBD) cDNA (amino acids 171–441) inserted into pET28a vector (Novagen, USA) was heterologous expressed in* Escherichia coli* BL21(DE3) strain. The protein was purified in buffer A (20 mM HEPES pH 7.5, 300 mM NaCl, 5% glycerol) onto a Talon Superflow Metal Affinity Resin (BD Biosciences Clontech, Palo Alto, CA), according to the previously described protocol [5].

### 2.7. Thermal Shift Assay (TSA)

This assay was performed following qualitative and quantitative approaches. Qualitative TSA used 10 *μ*M of hPPARß/*δ* LBD, 5x SYPRO® Orange (Sigma Aldrich) in buffer containing 20 mM HEPES pH 7.5, 200 mM NaCl, and 5% glycerol, and 30 *μ*M (~0.01 *μ*g/mL) of commercial agonists (1 protein : 3 ligand) or 140 *μ*g/mL of extracts from the tested library were added in a Microamp 96-well plate (Applied Biosystems). Quantitative TSA was performed in same conditions, varying ligand/extract concentrations (ligand concentrations were 0.3, 0.5, 1, 3, 5, 10, 30, and 50 *μ*M; extract concentration were 5, 10, 30, 50, 100, 300, and 500 *μ*g/mL). The experiment was performed at 7500 PCR Real Time System (Applied Biosystems), and measurements were taken from 9°C to 89°C, with a gradient of 1°C per minute, totalizing 80 measurements [23]. The assay was performed in triplicate and data were analyzed at OriginPro8.1. For Kd determination data were fitted using Hill1 model (OriginPro 8.1).

### 2.8. ANS Fluorescence Quenching

The conditions for 8-anilinonaphthalene-1-sulfonic acid (ANS) assay was optimized in terms of protein and ANS concentrations (Supplementary Material Figure 1), in order to define if ANS quenching was really promoted by PPARß/*δ* ligand binding. In a 96-well black microplate (Greiner Bio-One), 2 *μ*M hPPARß/*δ* LBD and 20 *μ*M ANS were incubated in 20 mM HEPES pH 7.5, 200 mM NaCl, and 5% glycerol, at 4°C. After 1 hour, the compounds or extracts were added to the mixture. For the commercial agonist test, 0.3 *μ*M (0.0001 *μ*g/mL), 0.5 *μ*M (0.0002 *μ*g/mL), 1 *μ*M (0.0004 *μ*g/mL), 3 *μ*M (0.001 *μ*g/mL), 5 *μ*M (0.002 *μ*g/mL), 10 *μ*M (0.004 *μ*g/mL), 30 *μ*M (0.01 *μ*g/mL), and 50 *μ*M (0.02 *μ*g/mL) concentrations were used; for the extracts, 5 *μ*g/mL, 10 *μ*g/mL, 30 *μ*g/mL, 50 *μ*g/mL, 100 *μ*g/mL, 300 *μ*g/mL, 500 *μ*g/mL, and 1 mg/mL concentrations were used. The assay was read on EnSpire Multimode Plate Reader (Perkin Elmer) with 380 nm excitation and emission scanning between 400 and 600 nm, at 25°C. The maximum fluorescence emission intensities (480 nm) were plotted versus each compound/extract concentration for affinity constant calculation, at OriginPro 8.0, through Hill1 sigmoidal adjust.

### 2.9. Natural Extract Library

PPARß/*δ* primary screening was performed against 560 hydroalcoholic extracts from the Phytobios library. The Phytobios library was kindly provided by Chemistry of Natural Products Library (LQPN) from the Brazilian National Bioscience Laboratory (LNBio/CNPEM) in partnership with Phytobios Ltda, which planned and assembled the library. The Phytobios/LNBio library regularly has extracted plant samples from Amazonian forest, Atlantic forest, Cerrado, and Caatinga. Each sample is accompanied by precise collecting location by GPS; plant identification by a qualified botanical taxonomist; and a deposit of testimony exsiccate in a certified herbarium. Each collection gets at least 5 kg of leaves (and/or roots and or barks). After processing, each sample gives about 20 g of dry extract, enough for many test repetitions and each sample is fractionated in 9 (nine) chromatographic fractions and immediately plated in 384 wells plates and frozen for further assays. Therefore 10 (ten) samples = 9 fractions + the crude extract are available for testing. This processing allows access to low concentration and yet unknown bioactive substances that are generally hidden by the majoritarian substances. All samples were submitted to analysis by mass spectrometry + molecular networking technique (data not shown). The tested library contained 560 hydroalcoholic extracts from Brazilian plants assembled in two 384 microplates. The compounds were preplated in 384 well microplates at the stock concentration of 10 mg/mL, in 100% DMSO. Before screening, compounds were transferred to daughter plates and diluted to 1 mg/mL, in 100% DMSO. Columns 1, 2, 23, and 24 from daughter plates were empty, and the positive and negative controls were filled in the screening plates.

## 3. Results

### 3.1. Optimizing the Screening Conditions

**Figure 2:**
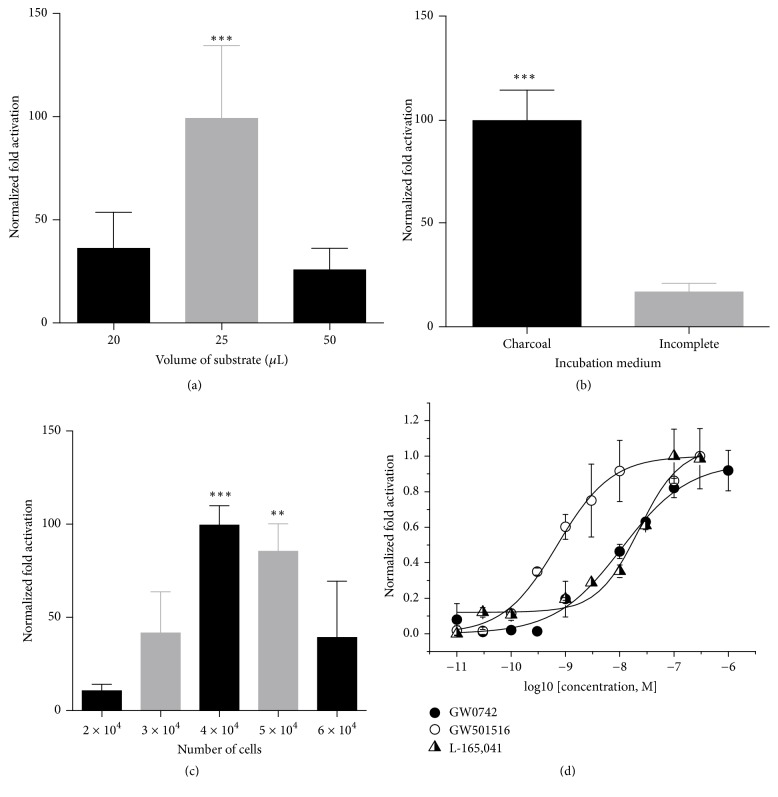
Optimization of the screening protocols. When not specified, the positive control was 1 *μ*M of GW0742 and the number of cells was 4 × 10^4^ cell/well. Bar graphs represent the specific fold activation normalized by the highest activation presented as the mean ± SD. *p* values were calculated by the unpaired *t*-test (^*∗∗*^*p* < 0.01 and ^*∗∗∗*^*p* < 0.001). All bar graphs and calculations were performed with GraphPad Prism. (a) Evaluation of substrate volumes. Cell lysis was performed in 20 *μ*L of passive lysis buffer, and the volumes of LAR II and Stop & Glo substrates were varied (50, 25, and 20 *μ*L). The results show that the best signal of PPARß/*δ* activation was measured with 25 *μ*L of each substrate. The data is from one experiment with 6 technical replicates. (b) Evaluation of the best ligand incubation medium. In this test, 293T cells were incubated with GW0742 in 10% charcoal-stripped FBS DMEM (charcoal) or in DMEM (incomplete), and the activation fold of PPARß/*δ* was measured in each condition. *Z*′-factor charcoal = 0.62; *Z*′-factor incomplete = 0.21. Charcoal-supplemented medium was chosen as the best option for our assays, and this medium composition was used in the ligand screening for PPAR*β*/*δ*. One representative experiment is shown out of three independent replicates. (c) Evaluation of the number of cells per well. Cells were seeded at 2 × 10^4^, 3 × 10^4^, 4 × 10^4^, 5 × 10^4^, and 6 × 10^4^ cells per well, and the activation fold of PPARß/*δ* was measured in each condition to determine the best quantity of cells in each assay. Based on these results, we chose 4 × 10^4^ cells per well to perform PPARß/*δ* ligand screening. One representative experiment is shown out of four independent replicates. (d) PPARß/*δ* dose-response activation in the presence of the commercial agonists (GW0742, GW501516, and L-165,041). Concentrations varied from 10^−11^ to 10^−6 ^M. Data are expressed as the normalized activation fold as 1 (maximum activation) and 0 (vehicle-treated cells) and represent the mean of 2 independent experiments with 3 technical triplicates. Graphs and dose-response calculations were performed in OriginPro 8.0. GW0742: *R*^2^ = 0.98549 and EC_50_ = 1.08708*E* − 8; GW601516: *R*^2^ = 0.98028 and EC_50_ = 7.10385*E* − 10; L-165,041: *R*^2^ = 0.9725 and EC_50_ = 2.23969*E* − 8.

Here we measured the PPARß/*δ* activity in transactivation assays under different circumstances with the goal of determining the best screening conditions. This screening setup included the evaluation of luciferase substrate volumes, the medium for drug-incubation, and the cell number per well (Figure 2).

First, we tested different volumes of the Dual Luciferase Assay Reporter System components to define the best signal-to-noise ratio without harming the assay quality. The solution volumes recommended by the manufacturer are 100 *μ*L of luciferase substrate per well. However, we verified that 25 *μ*L of each substrate is sufficient to provide a high signal with good discrimination between the activated and nonactivated PPARß/*δ* (Figure 2(a)). In this way, we reduced the cost and reagent usage by 75% without losing signal information. This reduction represents a major decrease in the cost of high- and medium-throughput assays, as these campaigns usually screen hundreds and thousands of compounds at the same time.

Another important verification was related to the definition of the best medium composition used in the assay that improved the data quality. Since natural fatty acids work as PPARß/*δ* natural ligands and FBS contains many of these natural fatty acids, 10% FBS-supplemented DMEM may not be suitable for PPARß/*δ* agonist screening [15, 16, 18]. To overcome this limitation, we tested different medium compositions during compound/extract incubation. Our results showed that serum-free DMEM was considered inadequate since GW0742 activation was low, and the calculated *Z*′-factor of tested plate was below the reliability limit (*Z*′-factor = 0.21) [22] (Figure 2(b)). On the other hand, the assay performed with 10% FBS charcoal-stripped-supplemented DMEM showed a higher agonist-activation fold and better *Z*′-factor (0.56). Due to these results, the 10% FBS charcoal-stripped-supplemented DMEM was selected as the incubation medium for PPARß/*δ* agonist screening assays.

Interestingly, instead of HeLa or Cos-1, we chose the 293T cell lineage, which had not yet been described for PPARß/*δ* screening assays. This lineage is easy to cultivate, grow, and transfect as well as being one of the most industrially relevant cell lines due to the fact that it is cGMP compliant [24]. Additionally, we checked different concentrations of cells per well in the range (10,000–40,000) previously described for other cellular types [15, 17, 19]. Our results showed PPARß/*δ* activation of 199-fold when 40,000 cells were seeded per well (Figure 2(c)). Therefore, this quantity was selected due to its higher activation and small deviation.

In summary, we standardized that the best conditions for running our PPAR*β*/*δ*-screening assay use 40,000 transfected cells per well and incubation in 10% FBS charcoal-stripped-supplemented DMEM and with a 75% reduction of luciferase substrates (25 *μ*L of both the luciferase substrates LAR II and Stop & Glo®).

### 3.2. Sensitivity of the Assay against Known Agonists

To verify the sensitivity of our assay, we measured the PPARß/*δ* activation under treatment with its commercial agonists GW0742, GW501516, and L-165,041 in dose-response curves (10^−11^–10^−6 ^M) (Figure 2(d)). These ligands are pure compounds known to induce high cellular transactivation of PPARß/*δ* [5, 7, 25]. By our results, we calculated the following EC_50_ for each tested compound: EC_50_ _GW501516_ = 0.71 nM, EC_50_ _GW0742_ = 10.87 nM, and EC_50_ _L-165,041_ = 26.40 nM on the same nanomolar scale found in the literature (EC_50_ _GW501516_ = 1.8 nM [7], EC_50_ _GW0742_ = 1–3.5 nM [5], and EC_50_ _L-165,041_ = 125 nM [25]). These results confirm that the proposed transactivation assay is robust enough to discriminate low activation signals from possible hit candidates, as it is capable of identifying signals from commercial agonists in concentrations lower than 1 nM.

### 3.3. *Renilla *Reporter Expression as an Indicator of Cytotoxicity

**Figure 3:**
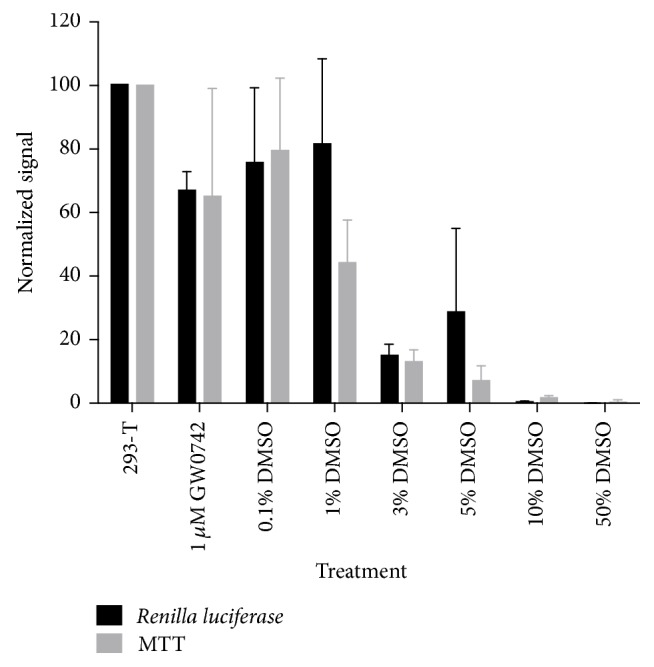
Development of a cytotoxic parameter. Comparison between the* Renilla *reporter expression with the MTT cytotoxicity signal in different DMSO concentrations (0.1%, 1%, 3%, 5%, 10%, and 50%) in order to validate the* Renilla *reporter expression as an indirect cytotoxicity parameter. One micromolar GW0742 diluted in DMSO, with a final concentration of 1% DMSO, was used as the positive control in the screening. Statistical analyses proved that our indirect cytotoxic parameter (*Renilla *reporter expression) can be used to identify cytotoxic compounds as well as the MTT cytotoxicity assay. Through analyzing the 2-way ANOVA statistics, there is no statistical difference (ns) between the two types of experiments, and the effect of the DMSO concentration was considered extremely significant according to GraphPad Prism. Followed by Sidak's multiple comparison test, the toxicity increases in concentrations above 3% of DMSO with statistical significance. Data are the mean ± SD (*n* = 4 independent replicates).

*Renilla *reporter expression is commonly used as a control for the transfection efficiency [20, 21]. Here, we propose using* Renilla *reporter expression as a parameter for indirect cytotoxicity. Since cells were transfected in a batch prior to plating in the screening plates,* Renilla *reporter expression among wells should be on the same order of magnitude among wells and decreases in this signal should indicate cytotoxicity [21]. The concentration of GW0742 (1 *μ*M, 1% DMSO as vehicle), used as a positive control in the screening, had no statistical difference in comparison with 0.1% or 1% DMSO (concentration used in our negative control), showing that GW0742 has no cytotoxicity. Using toxic concentrations of DMSO (3–50%), we demonstrated that analyses of the* Renilla *reporter expression had the same outcome as the MTT cytotoxicity experiment (Figure 3); that is, we can imply, with statistical significance (*p* < 0.0001), that compounds or extracts that led to low* Renilla *reporter expressions also resulted in high cellular toxicity. Therefore, we defined low* Renilla *reporter expression as an indirect cytotoxicity parameter of our assay, and in further screenings, compounds/extracts that led to low transfection signals were disregarded. In this manner, in one transactivation assay, we obtained two types of different results: the primary firefly reporter, which indicated PPARß/*δ* activation, and a second control with* Renilla* luciferase to detect the cytotoxicity.

### 3.4. Transactivation Screening with a Real Library

**Figure 4:**
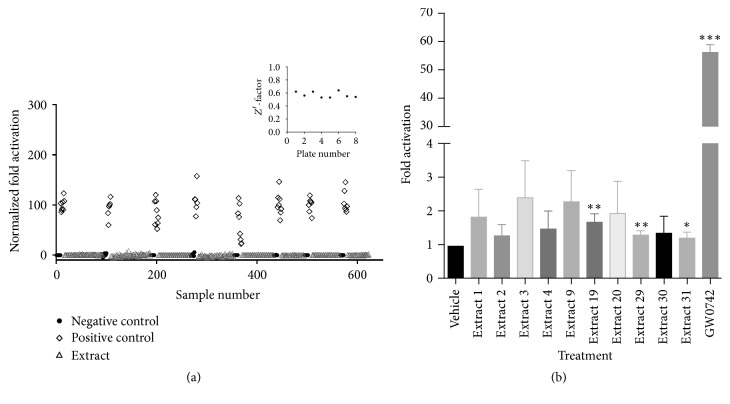
High-throughput screening assay and statistics. (a) HTS results are expressed by the* firefly/Renilla *ratio of each compound normalized by positive and negative controls, which were set as 100% and 0%, respectively, for each plate. It is possible to observe that the positive control varied among different plates and wells. Despite the fact that the searched extracts presented low PPARß/*δ* activation, they still presented significant differences in comparison to the negative controls (signal 7 times higher than the negative control standard deviation). Insert: *Z*′ values for each screened plate presented the high reliability of the data. (b) Confirmatory transactivation assay in triplicate using the possible hit candidates from the previously screened Phytobios library with 1 *μ*M GW0742 as the positive control, 1% DMSO as the negative control (vehicle), and 0.01 mg/mL of the tested extracts. We considered confirmed hit candidate extracts that showed* firefly/Renilla *ratios seven times higher than the standard deviation (>7 × DP) for the negative control treatment for at least two of the three replicates. Data are the mean ± SD. *p* values were calculated by the unpaired *t*-test (^*∗*^*p* < 0.05, ^*∗∗*^*p* < 0.01, and ^*∗∗∗*^*p* < 0.001) with GraphPad Prism.

After optimization of the cell-based transactivation assay with well-known commercial agonists, we submitted this assay to one natural extract library (Phytobios library). Our results showed that most of the extracts/fractions and negative controls presented low firefly luciferase expression and, therefore, a low* firefly/Renilla* ratio (Figure 4(a)). On the other hand, treatment with the positive controls presented high firefly luciferase expression and a high* firefly/Renilla *ratio, as expected.

After our data analysis, we found 31 possible hit candidates for PPARß/*δ* agonists (extracts 1–31), which showed activation rates from 1.3- to 2.1-fold. However, the obtained signal was much lower than the ones obtained in positive control treatment. These results could be explained by the fact that GW0742 is a commercial agonist with high specificity and affinity for PPARß/*δ* [5]. This means that this ligand has already been submitted to optimization steps through lead generation, while the Phytobios library is composed of raw plant extracts, which are a mixture of different compounds in different concentrations that need further fractionation and improvement. For all screening plates, we obtained an appropriate *Z*′-factor higher than the 0.5 limit (0.53–0.64) [26], indicating that our assay is reliable and suitable enough for PPARß/*δ* agonist screening (Figure 4(b)). The variability of PPARß/*δ* activation by the positive control (GW0742), even though the same batch of transfected cells, culture medium, agonist aliquot, and reading solutions was used, did not interfere in the *Z*′-factor assessment and was considered intrinsic to the experiment.

Next, to confirm the selected hit candidates, we performed a secondary transactivation screening. After this confirmatory screening, our results presented 10 possible hit candidates (extract 1, extract 2, extract 3, extract 4, extract 9, extract 19, extract 20, extract 29, extract 30, and extract 31), with activation rates from 1.2- to 2.4-fold (Figure 4(b)). When compared with GW0742 PPARß/*δ*-activation (56-fold), all fractions showed a much lower signal, but the signals were still above our selection criteria based on the standard deviation of negative controls. Moreover, as was mentioned above, the library contains raw plant extracts, which are a mix of diverse compounds in different concentrations, and probably, the compounds that activate PPARß/*δ* are present in very low amounts. In this context, the low PPARß/*δ* activation rates found with the extracts should be considered to be very significant.

### 3.5. Qualitative TSA Worked as a Confirmatory Assay for PPARß/*δ* Structure Stabilization by the Hit Candidate

The qualitative TSA was one additional validation methodology of our screening, measuring the tertiary structure stabilization of the PPARß/*δ* before and after ligand binding. As it was reported, NR ligands increase NR structural stability mainly because the ligand binding organizes specific interactions in their LBD pocket, which raises the degree of solvent protection and therefore, makes their structure more rigid [27–30].

**Figure 5:**
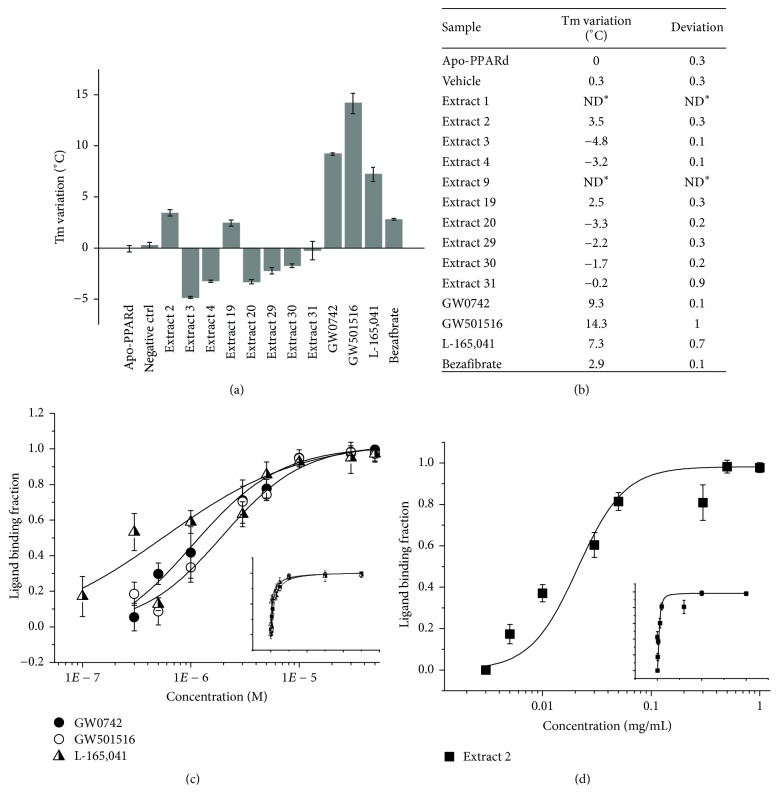
Validation assays of PPARß/*δ* ligand screening. (a) Thermal shift assay of hit candidates. Ten micromolar PPARß/*δ* with commercial agonists (GW0742, GW501516, L-165,041, and Bezafibrate) at 30 *μ*M or extracts at 0.14 mg/mL. The vehicle is DMSO. Experiments were performed in triplicate. Data are the mean ± SD. (b) Table with *T*_*m*_ variation and standard deviation for the extracts/compounds from the thermal shift. The experiment was performed in triplicate. ND^*∗*^: not defined. It is possible to verify that two possible hit candidates stabilized the receptor structure by more than 2.5°C, indicating direct binding to the receptor. (c)-(d) Dissociation curves for PPARß/*δ* ligands and hit candidates. Normalized fluorescence intensity at the emission maximum (480 nm) versus the ligand/fraction concentration, adjusted by the Hill1 approach with OriginPro 8.0. Dissociation curves for the commercial agonists varied from 10^−7^ to 10^−5^ *μ*M. In the dissociation curve, the concentration varied from 0.003 to 1 mg/mL. Data are the mean ± SD (*n* = 3 independent replicates).

Here, we first tested commercial agonists; our results showed that this technique is able to discriminate among specific agonists, not-specific agonists, and apo-PPARß/*δ* (Figures 5(a)-5(b)). The specific agonists (GW0742, GW501516, and L-165,041) lead to an increase in the protein melting temperatures (*T*_*m*_) in comparison to the apo-PPARß/*δ*  *T*_*m*_, indicating tertiary structure stabilization (Figure 5(a)). In particular, the GW0742 agonist stabilizes the tertiary structure of PPARß/*δ*, as was previously reported [5], increasing its *T*_*m*_ by 9.3 ± 0.1°C. The other specific agonists, GW501516 and L-165,041, also increased the *T*_*m*_ values of PPARß/*δ* by 14.3 ± 1°C and 7.3 ± 0.7°C, respectively. Bezafibrate, a PPAR pan-agonist with very low specificity to PPARß/*δ* that provides low activation [31], presented a *T*_*m*_ increase of only 2.9 ± 0.1°C, suggesting that the assay is sensitive to evaluate low-specificity hit candidates that might appear during compound screening.

In parallel, the TSA results of the hit candidates showed that 2 selected fractions (extract 1 and extract 9) did not shown the expected melting curves, indicating that these extracts somehow might destabilize the tertiary structure of PPARß/*δ* or even not directly bind to this receptor. On the other hand, 2 fractions (extract 2 and extract 19) increased the PPARß/*δ*  *T*_*m*_ by 3.5 ± 0.3°C and 2.5 ± 0.3°C, respectively, in comparison with the *T*_*m*_ of apo-PPARß/*δ* (Figure 5(a)). This result suggests that these extracts may have components that physically bind to the receptor and promote the stabilization of the protein structure.

### 3.6. ANS Fluorescence Quenching Determines the Affinity of the Hits in the Ligand Binding Pocket of PPARß/*δ*

The third experiment of this pipeline is the ANS fluorescence quenching assay, which determines the affinity of the selected hit candidate in the PPARß/*δ* hydrophobic binding site. In this assay, the ANS probe binds to the hydrophobic ligand binding pocket (LBP) of PPARß/*δ*, and it can be displaced by PPARß/*δ* ligands, causing fluorescence quenching (Supplementary Figure 1). As the agonist concentration increases, the fluorescence quenching becomes higher. Several tests were performed to evaluate the best probe: protein ratio for the best assay performance (Supplementary Figure 1). We also made PPARß/*δ*-ligand/extract binding curves with a 1 : 1 (probe : protein) stoichiometry, which showed that these molecule/extract ratios were effective in dissociating ANS from the PPARß/*δ* binding site, even in unsaturated ANS concentrations (Supplementary Figure 2). Finally, after all of the performed tests, we standardized the experiments with a 5-fold excess of ANS (Figures 5(c)-5(d)) to guarantee that all of the PPARß/*δ* is saturated by ANS and all of the conformational modifications caused by ligands/extracts in the receptor's LBP will provoke ANS probe displacement. After that, we measured and calculated the apparent dissociation constants (Kd_app_) of PPARß/*δ* bound to commercial agonists GW0742 (1.2 ± 0.3 *μ*M), GW501516 (1.8 ± 0.1 *μ*M), and L-165,041 (1.09 ± 0.08 *μ*M) (Figure 5(c)). Bezafibrate did not dislocate the ANS probe, with a behavior similar to the negative control (vehicle, DMSO) (Supplementary Figure 2), which is explained by its low specificity and affinity for PPARß/*δ* [31]. This result means that our pipeline is sensitive enough to evaluate low-specificity hit candidates. However, it is not possible to determine the Kd of this type of candidate. Finally, our results showed an apparent dissociation constant (Kd_app_) of 0.022 ± 0.008 mg/mL for the hit candidate extract 2 (Figure 5(d)), which is very close to the concentration used in the cellular transactivation assay (0.01 mg/mL).

### 3.7. Quantitative TSA Also Allows the Calculation of the Dissociation Constant

**Figure 6:**
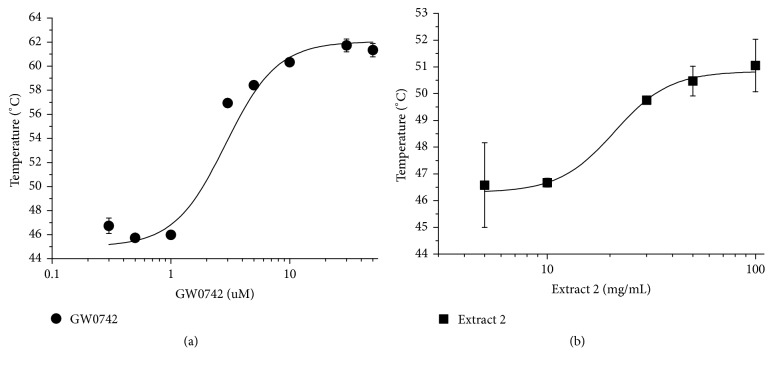
Quantitative TSA of PPARß/*δ* in different concentrations of GW0742 (a) and extract 2 (b). The curves were made using 10 *μ*M PPARß/*δ*. The GW0742 concentrations were 0.3, 0.5, 1, 3, 5, 10, 30, and 50 *μ*M, and the extract 2 concentrations were 5, 10, 30, 50, and 100 *μ*g/mL (higher concentrations of the extract were discarded since they absorb in the used wavelengths). The temperature was varied from 20°C to 90°C in a ramp of 1°C/minute. The *T*_*m*_ variations in the entire curves were approximately 16°C for GW0742 and 5°C for extract 2. Data are the mean ± SD (*n* = 3 independent replicates).

To confirm the apparent dissociation constant calculated by the ANS quenching assay, we conducted a quantitative thermal shift assay using increasing concentrations of GW0742 (positive control) and extract 2. To our knowledge, this is the first time that an ANS quenching assay was performed to characterize the binding affinities between the PPARß/*δ* binding pocket and ligands. Therefore, we submitted the extract and the commercial ligand to a more established protocol for the calculation of the dissociation constant [32]. By our results, we obtained a Kd_app_ of 20 ± 3.7 *μ*g/mL for extract 2 and a Kd_app_ of 2.6 ± 0.2 *μ*M for GW0742, which are very close and on the same order of magnitude as the ones obtained by the ANS quenching assay. In this way, we confirm that both the ANS quenching assay and TSA can be used for PPARß/*δ* dissociation constant evaluation, which present reliable results (Figure 6).

## 4. Discussion

The purpose of our study was to delineate a pipeline to search and characterize PPARß/*δ* agonists through a faster and cheaper transactivation primary screening, followed by two biophysical methods, aiming to exclude false positives and select molecules or extracts that directly bind and activate PPARß/*δ*.

The choice of the cellular transactivation reporter-gene assay as the first step in this pipeline enables the screening to start from a more physiological point of view [33, 34]. In this case, the selected molecules or extracts must permeate the cellular membranes, find and bind to the receptor, and promote its activation. Although other methods, such as TSA, ANS, and FRET, have been proposed to evaluate NR ligand binding [35–37], we consider that the transactivation assay produces quantitative and functional information in a short period of time, which makes it one of the most relevant and important assays for compound screening and drug discovery applied to NRs [33, 34]. Meanwhile, although* in vitro* FRET is the easiest to set up with commercial kits, it does not correlate with cellular conditions [37, 38]. ANS fluorescence quenching is also cheaper; nevertheless, it is laborious and time-demanding for HTS screening, beyond the fact that it is an* in vitro* approach [36, 39]. Moreover, even though TSA is designed to be applied in ligand screening [35, 40], it does not consider the intrinsic fluorescence of natural extracts or the high hydrophobicity of PPARß/*δ* LBD, which may interfere with the fluorescence signal. In summary, TSA, ANS, and FRET share the disadvantages of biophysical assays as they do not always correlate well with in vivo studies [34].

In summary, we suggest that transactivation reporter-gene assays in cell culture are the most verisimilar assays, as they exploit the natural signaling pathway of NRs; when ligands are added to the system, the receptor is activated and there is the consequent production of reporter protein, which can be measured [33]. Therefore, biophysical methods can and should be used as additional steps of screening pipelines, as they give important information for hit characterization like direct binding confirmation (TSA) and dissociation constant evaluation (ANS and TSA). In comparison with FRET and Lantha-Screen, which may be considered cheaper than commercial kits, these chosen validation methods present the disadvantage of providing indirect results with coactivator measurements [41–43].

After extensive investigation, we established a 3-day transactivation assay, which is a reduction of 1 to 2 days in length in comparison with other transiently transfected cell assays [15–17]. We also optimized the incubation medium (10% charcoal-stripped FBS-supplemented DMEM) and cellular concentration (40,000 cells/well) for our experiment. The major improvement was the 75% reduction in the luciferase substrate volume, which represents a 75% reduction in the kit usage as well as cost, and it brings innovation and advantages when compared with the other transactivation assays in 96-well plates [15–17, 19].

Several reporter-gene screenings for NRs in general have been described as efficient and fast ways to obtain NR physiological responses in high-throughput screening [18, 44–47]. Regarding PPARß/*δ* assays, most of them use transient transfection taking one or two days longer than our proposed method [15–17], with just one exception, which is based on permanent gene reporter transfect cells [19]. Our reduction in the assay length represents decreased costs for screening campaigns. Furthermore, we found in some reports individually made transfections in each well of the microplate [16], and we consider that this approach cannot confirm if all of the wells were equally transfected and received the same amount of DNA. Following other HTS screening assays [15, 17, 18], we chose to perform transfection in a batch prior to plating the cells, as we considered that the cells would be more homogenously transfected, with all cells contained in the well submitted to the same treatment.

Another special detail in our screening assay is the fact that we chose a 1 *μ*M concentration for the positive controls. Although the EC_50_ values for most agonists used (GW0742, GW501516, and L-165,041) are in the nanomolar range, the majority of transactivation assays and screenings for PPARß/*δ* use a range between 0.1 and 40 *μ*M of commercial agonists as a positive control [16, 17, 19, 48]. In addition, as we showed, the proposed transactivation assay is sensitive enough to detect PPARß/*δ* agonists in concentrations varying from 10^−11^ to 10^−6 ^M, as we obtained the following EC_50_ values for the commercial agonists: EC_50_ _GW501516_ = 0.71 nM, EC_50_ _GW0742_ = 10.87 nM, and EC_50_ _L-165,041_ = 26.40 nM. In this way, our results indicate that our assay can be used to detect, at a low level, an agonist that activates PPARß/*δ*.

Following the sequence of our pipeline, two biophysical methods (TSA and ANS fluorescence quenching) were employed to characterize PPARß/*δ* ligand binding along with Kd evaluation. Several studies had shown that a ligand-NR complex has an increased structural stability in comparison to its apo form [27–30]. Qualitative TSA results provided measurement of the PPARß/*δ* LBD structural stability in the absence or presence of commercial ligands, and the results were able to discriminate between high affinity (GW0742, GW501516, and L-165,041) and low affinity (as the pan-PPAR agonist Bezafibrate) PPARß/*δ* ligands [5, 7, 25, 31]. Furthermore, the ANS quenching assay and quantitative TSA evaluated the selected compounds/extracts bound to hPPARß/*δ* LBD, providing dissociation constant values. Few studies show affinity constants between the NRs and their ligands, and most of them are based on cellular dose-response assays, which calculate indirect constant affinities [7, 25]. Here, we show an improvement in ligand binding characterization methods, using an ANS fluorescence quenching assay and quantitative TSA, which are able to evaluate the affinities of compounds/extracts that bind to the PPARß/*δ* LBD pocket. These approaches for PPARß/*δ* ligand characterization were compared themselves, and the apparent dissociation constants found in both methodologies were in the same range, increasing the data reliability of our Kd evaluations. Finally, application of these methodologies also has the advantage of measuring the relative activity of a compound (or a mixture of substances) without the requirement of prior information about the chemical structure of the ligand [36]. Therefore, we proposed that these methodologies are useful as additional steps in the screening of natural extract libraries.

As it has been extensively reported, natural extracts are good starting points to select compounds that may play important roles in treating or preventing human metabolic diseases or regulating physiological functions [49]. In addition, natural plant extracts could improve the chemical diversity of compounds, increasing the choices of finding new molecules with biological activity [50], especially in the case of libraries that explore particular biomes of Brazilian diversity. Studies have shown that one new focus in the treatment of metabolic syndromes is searching for novel agonists for PPARs from natural products, which present low toxicity and high efficiency [49, 50]. However, it is important to mention that the screening of natural extract libraries could result in low activity signals since each tested fraction/extract is composed of different compounds, and only one of them might present activity against a specific target. In this context, the measured activities tend to be smaller than the ones obtained from the positive controls, which are generally composed of one isolated compound [15, 17].

**Figure 7:**
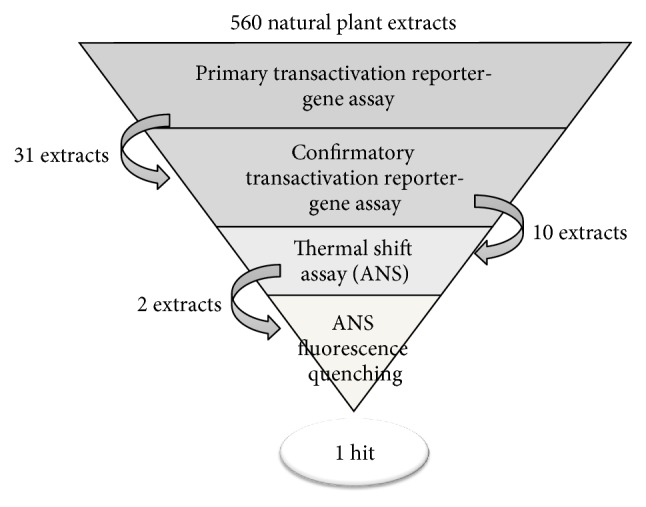
Screening pipeline for PPARß/*δ* agonists. Our proposed screening pipeline for PPARß/*δ* agonists is composed of 3 complementary assays. The primary transactivation reporter-gene assay screening and the confirmatory transactivation reporter-gene assay utilize a cellular transactivation reporter-gene assay that has been optimized to a 3-day long experiment with 40,000 cells and only 25 *μ*L of the luciferases substrates per well, reducing the time and cost of the screening assay. The two following validation assays are the thermal shift (TSA) to check if the compounds/extracts previously selected stabilize the PPARß/*δ* tertiary structure and the ANS fluorescence quenching to determine the compound affinity to the hydrophobic pocket of PPARß/*δ*. We submitted a 560-natural extract library to the proposed pipeline and found 31 possible hit candidates in the primary transactivation screening. Ten hit candidates were selected in the confirmatory cellular transactivation. The TSA selected 2 extracts, and one of them showed a 0.02 mg/mL affinity constant in the ANS quenching assay.

To test and best characterize our developed pipeline, we performed a validation screening with 560 natural extracts from the Phytobios library and found 31 possible hit candidates (Figure 7). All of the screening plates presented the statistical parameter *Z*′-factor values higher than the 0.5 limit (0.53–0.64) [22], indicating the robustness and reliability of this assay. The observed variation of the positive control activation fold among different plates was considered to be an intrinsic variability of the cellular assay, as it has been reported previously [5, 15, 17, 19, 48].

After confirming 10 hit candidates in a secondary transactivation assay, we started the selection of these extracts through the TSA and ANS assays, avoiding possible indirect and allosteric interactions. From the qualitative TSA results, two extracts have increased the receptor melting temperature, which means that they contain chemical components that bind and stabilize the tertiary structure of the receptor [27, 28]. However, qualitative TSA allows the selection of extracts with components that interact with other hydrophobic sites in the protein structure (besides LBD) [40]. To overcome this limitation, the ANS fluorescence quenching assay was applied to confirm the physical interaction between the compounds/extracts and PPARß/*δ* LBD, and it allows the evaluation of the apparent dissociation constants (Kd_app_). We selected the best extract from the library (extract 2), which binds PPARß/*δ* with a Kd_app_ of 22 ± 8 *μ*g/mL. Moreover, we performed an additional Kd_app_ evaluation, employing quantitative TSA. By using this technique, we obtained a Kd_app_ of 20.9 ± 3.7 *μ*g/mL for extract 2, showing the reliability of our Kd evaluations.

In addition, it is important to mention that the found apparent affinity constant (Kd_app_) for extract 2 is close to the concentration used in the cellular assay (0.01 mg/mL), which may explain the low-fold of activation (1.31-fold) found in the transactivation assay. Since extract 2 is a mixture of diverse chemical compounds, we suggest that at least one of its components provides PPARß/*δ* activation, binding to the receptor with a higher affinity. Thus, the use of higher extract concentrations would probably increase the degree of PPARß/*δ* activation. However, we observed that higher extract 2 concentrations were cytotoxic to cells (data not shown), and therefore, it would be interesting to fractionate this extract to concentrate and separate its bioactive compounds in order to decrease the cytotoxicity and possibly increase PPARß/*δ* activation.

## 5. Conclusion

In summary, we developed and validated a pipeline to screening for new PPARß/*δ* agonists in libraries of compounds or natural extracts. The first living cell screening gives information about the ability of the hit candidates to activate PPARß/*δ*. We optimized this assay in length (3 days long) and in the volume of reading reagents (75% reduction), which represents a real decrease in cost for screening campaigns. We also obtained information about the compound cytotoxicity, which adds an improvement in the obtained information from the primary screening. To exclude indirect activators of PPARß/*δ*, we joined two* in vitro* biophysics assays, creating a pipeline that searches for compounds/extracts that can activate, stabilize the tertiary structure, and bind to the hydrophobic pocket of PPARß/*δ*, allowing calculation of the apparent affinity constant. We screened a 560-natural extract library to test our pipeline and found 31 possible hit candidates in the primary cellular transactivation screening; from these, 10 hit candidates were selected in the confirmatory cellular transactivation, where 2 were selected by qualitative TSA, but only one was selected as a hit since it presented a real capacity to bind and activate PPARß/*δ* with a relatively high affinity. To date, our proposed pipeline presents more information than just a cellular activation screening, as it ranges from the cellular to the biophysical point of view, allowing the calculation of apparent affinity constants besides the traditional EC_50_ calculation. Moreover, we reduced the reagent use and time of the assay, which is relevant for big screening campaigns. Finally, this approach may improve the effectiveness of screening for agonists targeting PPARß/*δ* for drug development, with a significant reduction in the time and cost for the transactivation assay.

## Supplementary Materials

Supplementary 1 Supplementary Figure 1: tests of probe : protein concentrations for ANS fluorescence quenching assay. ANS quenching tests of probe and protein concentrations. hPPAR*β*/*δ* LBD concentrations varied from 0.25 to 2 *μ*M; ANS concentrations varied from 5 to 20 *μ*M. Protein-ANS mixtures were incubated for 1 hour at 4°C. After, GW0742 was added at 0.3 *μ*M, 0.5 *μ*M, 1 *μ*M, 3 *μ*M, and 5 *μ*M. DMSO was used as vehicle. The assay was read on the EnSpire Multimode Plate Reader (Perkin Elmer) with 380 nm excitation and emission scanning between 400 and 600 nm, at 25°C. The fluorescence emission intensities were plotted for each combination of protein and probe 9 10 11 12 13 14 15 16 concentrations in all emission wavelengths (400–600 nm). The chosen protein and ANS concentrations were 2 uM and 10 uM, which presented best signal-to-ratio noise without much reagent usage. In graphs 1–4 hPPAR*β*/*δ* concentration varied in the presence of 5 *μ*M ANS; 5–8, hPPAR*β*/*δ* concentration varied in the presence of 10 *μ*M ANS; 9–12, hPPAR*β*/*δ* concentration varied in the presence of 15 *μ*M ANS; and 13–16 hPPAR*β*/*δ* concentration varied in the presence of 20 *μ*M ANS.

Supplementary 2 Supplementary Figure 2: ANS quenching curves of PPARß/*δ* submitted to GW0742 (left) or extract 02 (right) titration. 10 *μ*M of hPPARß/*δ* LBD and 10 *μ*M of ANS were used. The found Kds (6.3 ± 1.3 *μ*M fo GW0742 and 113.9 ± 75 *μ*g/mL for extract 2) are probably overestimated, due to the fact that there are more than one possible ANS binding site in hPPARß/*δ* LBD, which are not necessary located into the LBP, but it is still possible to observe that both GW0742 and extract 02 dislocated ANS from PPARß/*δ* LBP. Data are the mean ± SD (*n* = 3 independent replicates).

Supplementary 3 Supplementary Figure 3: ANS fluorescence quenching assay for PPAR*δ* commercial agonist and hit candidate. 2 *μ*M hPPARß/*δ* LBD and 20 *μ*M ANS were incubated for 1 hour at 4°C. After, compounds or extracts were added to the mixture at 0.3 *μ*M, 0.5 *μ*M, 1 *μ*M, 3 *μ*M, 5 *μ*M, 10 *μ*M, 30 *μ*M, and 50 *μ*M (commercial compounds), or at 0.005 mg/mL, 0.01 mg/mL, 0.03 mg/mL, 0.05 mg/mL, 0.1 mg/mL, 0.3 mg/mL, 0.5 mg/mL, and 1 mg/mL (extracts). DMSO was used as vehicle (negative control). The assay was read on the EnSpire Multimode Plate Reader (Perkin Elmer) with 380 nm excitation and emission scanning between 400 and 600 nm, at 25°C. The fluorescence emission intensities were plotted for each compound/extract concentration in all emission wavelengths (400–600 nm). GW0742, GW501516, L-165,041, and the hit candidate extract 2 showed quenching in the ANS fluorescence. Bezafibrate had the same pattern as the vehicle (DMSO) and did not show a fluorescence quenching, meaning that the compound could not dislocate the ANS from PPARß/*δ* binding pocket.
