# Supplementary figures and images for: Cellular and Biophysical Pipeline for the Screening of Peroxisome Proliferator-Activated Receptor Beta/Delta Agonists: Avoiding False Positives
Source: PPAR Res. 2018 Apr 12;2018:3681590. doi: 10.1155/2018/3681590 (PMC5924986; doi:10.1155/2018/3681590)

**1**

**2**

**3**

**4**

**5**

**6**

**7**

**8**


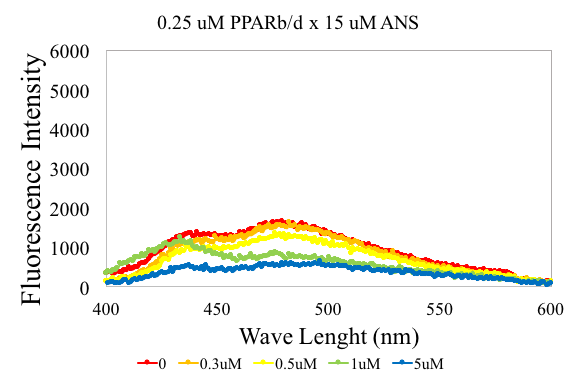

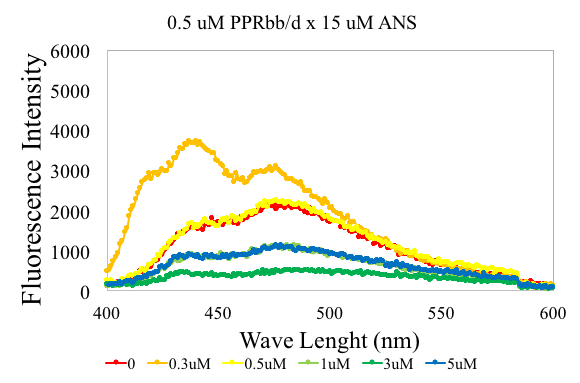


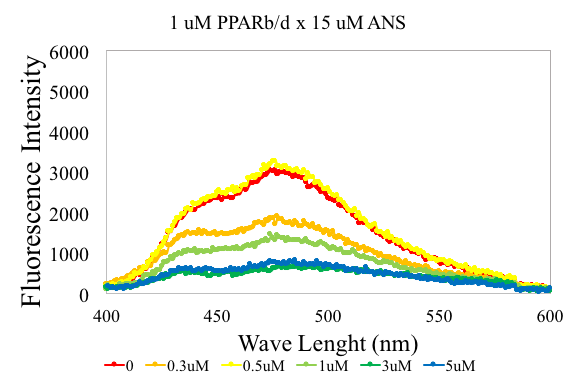

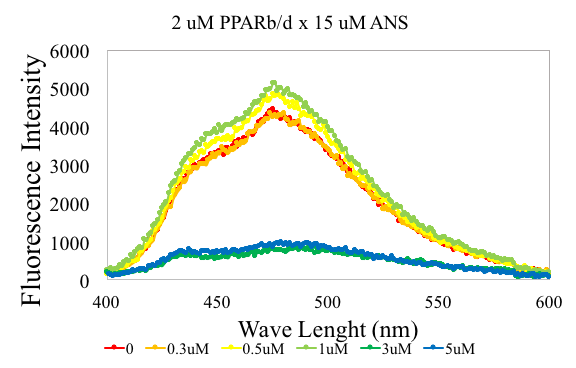


**9**

**10**

**11**

**12**

**13**

**14**

**15**

**16**

Supplement: Supplementary 1 — Supplementary Figure 1: tests of probe : protein concentrations for ANS fluorescence quenching assay. ANS quenching tests of probe and protein concentrations. hPPARβ/δ LBD concentrations varied from 0.25 to 2 μM; ANS concentrations varied from 5 to 20 μM. Protein-ANS mixtures were incubated for 1 hour at 4°C. After, GW0742 was added at 0.3 μM, 0.5 μM, 1 μM, 3 μM, and 5 μM. DMSO was used as vehicle. The assay was read on the EnSpire Multimode Plate Reader (Perkin Elmer) with 380 nm excitation and emission scanning between 400 and 600 nm, at 25°C. The fluorescence emission intensities were plotted for each combination of protein and probe 9 10 11 12 13 14 15 16 concentrations in all emission wavelengths (400–600 nm). The chosen protein and ANS concentrations were 2 uM and 10 uM, which presented best signal-to-ratio noise without much reagent usage. In graphs 1–4 hPPARβ/δ concentration varied in the presence of 5 μM ANS; 5–8, hPPARβ/δ concentration varied in the presence of 10 μM ANS; 9–12, hPPARβ/δ concentration varied in the presence of 15 μM ANS; and 13–16 hPPARβ/δ concentration varied in the presence of 20 μM ANS. [file 3681590.f1.docx]

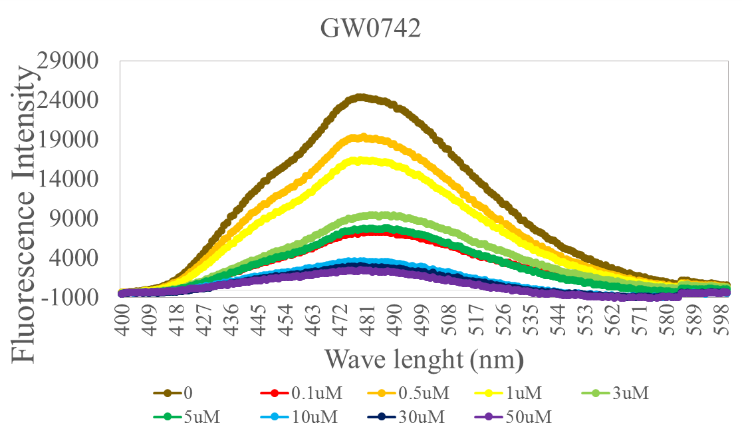

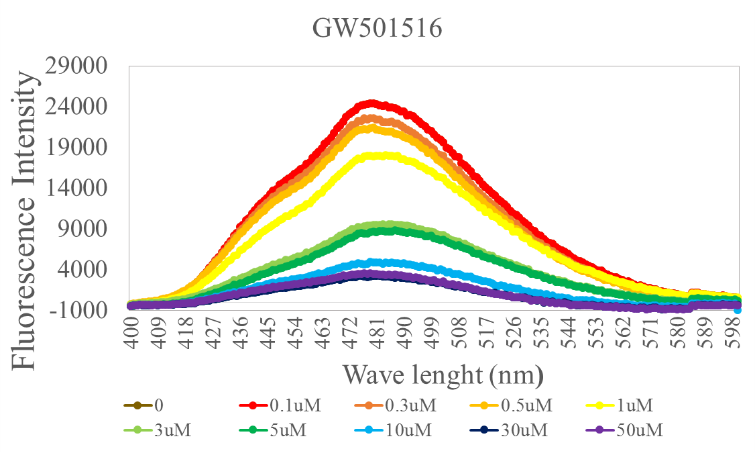


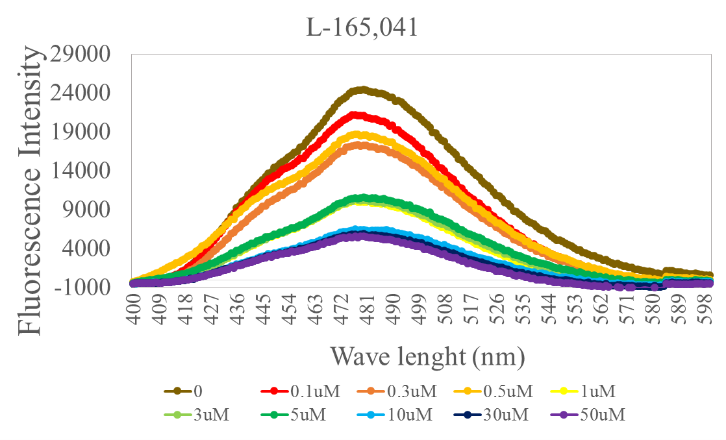

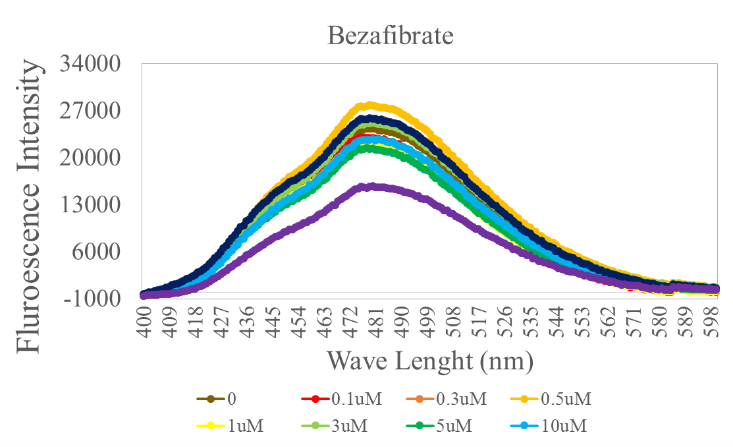

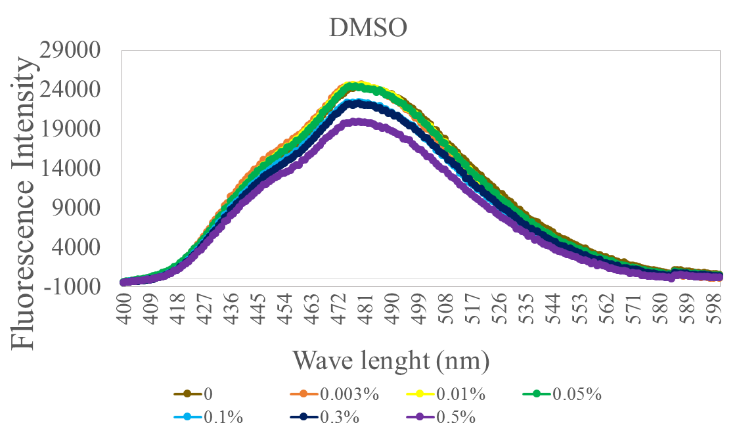

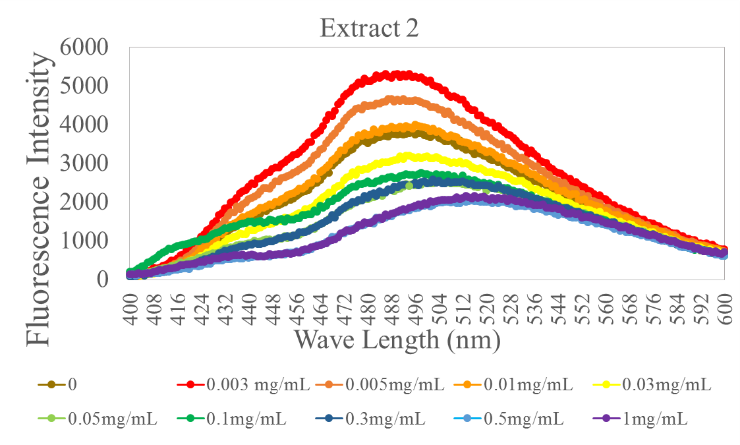

Supplement: Supplementary 3 — Supplementary Figure 3: ANS fluorescence quenching assay for PPARδ commercial agonist and hit candidate. 2 μM hPPARß/δ LBD and 20 μM ANS were incubated for 1 hour at 4°C. After, compounds or extracts were added to the mixture at 0.3 μM, 0.5 μM, 1 μM, 3 μM, 5 μM, 10 μM, 30 μM, and 50 μM (commercial compounds), or at 0.005 mg/mL, 0.01 mg/mL, 0.03 mg/mL, 0.05 mg/mL, 0.1 mg/mL, 0.3 mg/mL, 0.5 mg/mL, and 1 mg/mL (extracts). DMSO was used as vehicle (negative control). The assay was read on the EnSpire Multimode Plate Reader (Perkin Elmer) with 380 nm excitation and emission scanning between 400 and 600 nm, at 25°C. The fluorescence emission intensities were plotted for each compound/extract concentration in all emission wavelengths (400–600 nm). GW0742, GW501516, L-165,041, and the hit candidate extract 2 showed quenching in the ANS fluorescence. Bezafibrate had the same pattern as the vehicle (DMSO) and did not show a fluorescence quenching, meaning that the compound could not dislocate the ANS from PPARß/δ binding pocket. [file 3681590.f3.docx]
